# Machine Learning Classification Models with SPD/ED Dataset: Comparative Study of Abstract Versus Full Article Approach

**DOI:** 10.1007/978-3-030-51517-1_31

**Published:** 2020-05-31

**Authors:** Mayara Khadhraoui, Hatem Bellaaj, Mehdi Ben Ammar, Habib Hamam, Mohamed Jmaiel

**Affiliations:** 8 Digital Research Centre of Sfax, Sfax, Tunisia; 9 Institut Mines-Télécom, CNRS, Paris, France; 10 Université de Sherbrooke, Sherbrooke, QC Canada; 11 Digital Research Centre of Sfax, Sfax, Tunisia; 12 University of Sfax, Sfax, Tunisia; 13 ENIS, ReDCAD Laboratory, University of Sfax, B.P. 1173, Sfax, Tunisia; 14 Digital Research Center of Sfax, 3021 Sfax, Tunisia; 15 Solutions Galore Inc., Moncton, NB Canada; 16 Faculty of Engineering, Moncton University, Moncton, NB Canada

**Keywords:** Text classification, Data mining, Supervised machine learning, Medical informatics, Public health

## Abstract

In response to the researchers need in the bio-medical domain, we opted for automating the bibliographic research stage. In this context, several classification models of supervised machine learning are used. Namely the SVM, Random Forest, Decision Tree, KNN, and Gradient Boosting. In this paper, we conduct a comparative study between experimental results of full article classification and abstract classification approaches. Furthermore, we evaluate our results by using evaluation metrics such as accuracy, precision, recall and F1-score. We observe that the abstract approach outperforms the full article approach in terms of learning time and efficiency.

## Introduction

In the vast field of artificial intelligence, machine learning is called upon to play a central role allowing machines to learn automatically in the context of scientific research. In fact, the field of scientific research seems to be a challenging task and can generate difficulties for researchers. In this paper, we are interested in the epidemiological research domain. Here is a list of some of today’s challenges; 1) Research in medicine requires an efficient working methodology to better attain pertinent results, confirm/affirm or complete a hypothesis or theory, evaluate a procedure or a program, minimize bias, etc., 2) Medical researchers face challenges in epidemiological research, such as the choice of population, sample size, time of study, and target knowledge base; the selection of reference subjects; the required budget, data collection, 3) Developing coherent epidemiological research requires the integration of knowledge and skill, 4) Based on the results of [2], one of the major challenges of this specified domain is the literature review task which should be exhaustive. In this paper, we focus on the last aforementioned challenge. To overcome this problem, machine learning techniques and algorithms are recommended. In our work, we are concerned with several machine learning methods namely the Support Vector Machine (SVM), K Nearest Neighbor (KNN), Gradient Boosting (GB), Random Forest (RF), Decision Tree (DT), Multi-Nominal Naive Bayes (MNB) and Logistic Regression (LR).

The originality of our work lies in the creation of a new public database SPD/ED in the biomedical domain based on title, abstract, keywords and full scientific papers. Our database is a collection of several scientific papers classified into four different categories according to the taxonomy of the epidemiological studies (Analytic, descriptive, Meta-Analysis and Others) [5]. Based on the aforementioned machine learning methods, we will conduct a comparative study between the text classification task based on the abstract versus the full article.

The paper is organized as follows. Section 2 explains basic concepts of machine learning methods. Related work is discussed in Sect. 3. In Sect. 4, we present our method. Section 5 discusses the experimental results. Section 6 concludes the paper and outlines areas for future research.

## Machine Learning

In our work, we are interested on text classification, defined as the process of associating a category (or class) with free text, based on the information it contains, is an important element of information retrieval systems. In our work, we deal with the text classification challenge and accuracy problem. In fact, the main challenge consists in, for each new entry, being able to determine to which category this entry belongs. Associating a class with free text is a costly and difficult task, therefore the automation of this task has become a challenge for the scientific community. To help the scientific community the task of Text classification is assisted by the machine learning.

### Different Types of Approaches

There are several Machine learning methods: supervised, unsupervised, reinforcement and semi-supervised learning. In our work, we are interested on the supervised learning.

### Machine Learning Algorithms

The objective of machine learning is to recognize among data structures that are difficult to detect manually. From these structures, we seek to classify new textual data. In our work, we focus on the classification of scientific papers in the epidemiological domain based on the taxonomy of the epidemiological studies.

#### Decision Tree (DT)

Decision trees are classification rules which base their decisions on a series of tests associated with a set of attributes. These tests are organized in a tree structure. The internal nodes are called decision nodes. Each decision node is labeled by a test which can be applied to any description of an individual in the population.

#### Support Vector Machine (SVM)

Support Vector Machines is a phenomenon f (possibly non-deterministic) which, from a certain set of inputs x, produces an output ${\text{y}} = {\text{f }}({\text{x}})$. This approach, often translated by the name of Support Vector Machine (SVM), is a class of learning algorithms initially defined for discrimination and prediction of a binary qualitative variable. The main objective is to find f from the only observation of a certain number of input-output pairs $\{ ({\text{xi}},{\text{ yi}}){:}\,{\text{i}}\, = \, 1, \ldots ,{\text{n}}\}$. Among its advantages, SVM overcomes various common problems related to the recognition of shapes.

#### K-Nearest Neighbors (KNN)

The principle of this model consists in choosing the k data closest to the point studied in order to predict its value. The objective is to make a classification without making a hypothesis on the function ${\text{y}}\, = \,{\text{f }}\left( {{\text{x1}},{\text{x2}}, \ldots ,{\text{xn}}} \right)$ which links the dependent variable y to the independent variables ${\text{x1}},{\text{x2}}, \ldots ,{\text{xn}}$. Otherwise, the idea of the KNN algorithm is for a new observation (${\text{u1}},{\text{ u2}}, \ldots ,{\text{up}}$) to predict the k observations that are most similar to it in the training data [1].

#### Multi-nominal Naïve Bayes (MNNB)

The Multi-Nominal Naïve Bayes classifier is derived from Bayesian decision theory. It is a fundamental statistical approach in pattern recognition. Bayesian decision theory chooses the best decision among the possible decisions based on these laws and the costs associated with each decision. The objective consists in finding a decision rule which minimizes an average cost and in defining which decision (action) to take according to the observed entity.

#### Random Forest (RF)

The algorithm of “random forests” was proposed by Leo Breiman and Adèle Cutler in 2001 [3]. It performs parallel learning on multiple decision trees randomly constructed and trained on subsets of data different. The ideal number of trees, which can go up to several hundred or more, is an important parameter: it is very variable and depends on the problem.

#### Gradient Boosting (GB)

This boosting technique is mainly used with decision trees (it is then called Gradient Tree Boosting). Again, the main idea is to aggregate several classifiers together but to create them iteratively. These “mini-classifiers” are generally simple and parameterized functions, most often decision trees, each parameter of which is the split criterion of the branches.

## Related Work

In reference [5], the authors presented the various classic and new techniques for classifying texts: the preprocessing of documents such as tokenization, the removal of stop words, stemming; Lemmatizing, machine learning algorithms for document modeling; representation of document characteristics, optimal data representation; learning based on machine learning classifiers; measuring the performance of the classification model based on evaluation methods and performance metrics.

The authors of reference [5], presented five classifiers (SVM, NB, KNN, Decision Tree and Decision Table) with three different versions of the database. In addition, accuracy and scalability are calculated to evaluate and examine the advantages and disadvantages of them for Arabic TC based on the efficient tools of machine learning (Weka and RapidMiner).

In reference [4], the authors summarized the eminent multi-class classifiers, based on the literature, in order to apply them to evaluate on a new benchmark dataset of Vietnamese News (VNNews-01). In the data collect process, they are referred to more than thirty Vietnamese online newspaper websites and grouped into twenty-five categories. They added that their work might promote the text mining research in Vietnam.

Some authors present a comparative study of three machine learning algorithm to do the task of classifying human facial expression. Then, they analyzed the main performance. In the experimental study process, they introduced 23 variables calculated from the distance of facial features as the input in the classification phase. As output, they defined seven categories, such as: angry, disgust, fear, happy, neutral, sad, and surprise. As experimental results, they recorded 75.15% of K-Nearest Neighbor (KNN)’s accuracy, 80% for Support Vector Machine (SVM), and 76.97% for Random Forest algorithm. As for the result using the largest amount of data, the accuracy is 98.85% for KNN, 90% for SVM, and 98.85% for Random Forest algorithm [6].

## Method

### Data Collection

In our work, we focused on supervised learning. To do so, we collected a set of labeled scientific articles from different scientific journals including Science direct, PubMed, Google scholar, etc. In addition, scientific articles were classified in 4 different predefined classes related to the taxonomy of epidemiological study, including Descriptive, Analytic, Meta-Analysis and Experimental. The several categories’ definitions are presented in Table 1.

**Table 1.** Label encoding

Category code	Category name	Definition
0	Analytic study	Raise etiological hypotheses by comparing the prevalence of the event in exposed and unexposed subjects
1	Descriptive study	Describe phenomena and their geographic distribution and temporal
2	Meta-Analysis study	Assess the effect of a treatment used on comparable populations, by combining the results of multiple studies
3	Experimental study	Intervene on the exhibition status of subjects. It can affect the factor (s) of exposure, the time of exposure and the people exposed

**Fig. 1.**
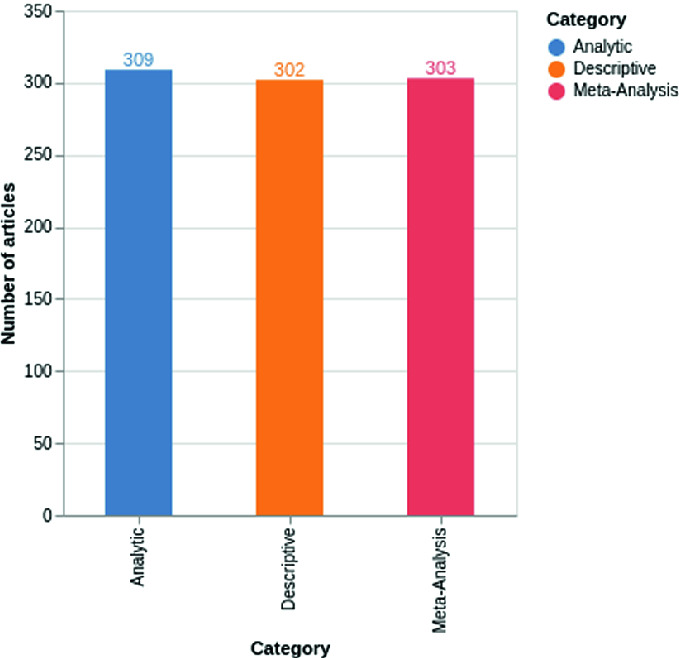
The distribution of articles across the different values of labels/article.

**Fig. 2.**
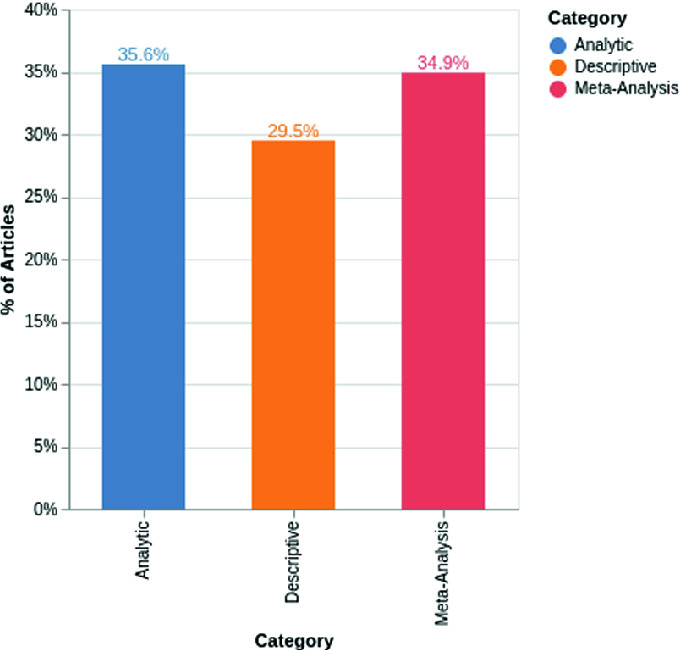
Histogram representation of the articles/label Percentage

Data collection was performed on the basis of two different approaches. The first approach is only interested in the Abstract part. We notice that, in the field of epidemiology, the Abstract part is composed of different parts in particular Aim/Introduction/Purpose, Methods, Results and discussion and conclusion. The first approach reveals a first database made up of 300 abstracts per category. The second approach is to collect the full article without omitting any section from Abstract to the references. This exercise led us to the creation of a second extended database of 300 articles by category. Figures 1 and 2 exemplify the distribution of scientific papers according to their categories.

It is worth noting, that this is a critical step since this task is normally performed manually. The state of the art of existing databases shows that there is no standard corpus containing scientific articles classified according to the taxonomy of epidemiological studies. Scientific papers are labeled according to different predefined classes according to the taxonomy of the epidemiological study. For that reason, in the data collect process, we were aware that the quality of data plays a vital role in the training data process and the calculation of the accuracy score of any machine learning classification algorithm. Based on the carefully selected data, the machine learning algorithms can learn the patterns and correlations in the data.

### Data Preprocessing

In order to transform raw data into an understandable format, we aim to apply data preprocessing techniques to build machine learning classifier. In fact, the data should be cleaned and preprocessed to eliminate characteristics of less important data and improve accuracy. For this purpose, we used machine learning techniques such as lowercasing which defines a common approach to reduce all the text to lower case for simplicity, Tokenization which assumes splitting text into tokens, Punctuation Removal which is a form of pre-processing to filter out useless data and Stop words Removal.

### Data Representation

The TF * IDF (for Term Frequency * Inverse Document Frequency) is the result of a calculation, in the algorithm of search engines, allowing to obtain a weight, an evaluation of the relevance of a document compared to a term, taking into account two factors: the frequency of this word in the document (TF) and the number of documents containing this word (IDF) in the corpus studied. The TF * IDF is expressed as follows:

$$ w_{i,j} = tf_{i,j} \times log\left( {\frac{N}{{df_{i} }}} \right) $$

Where *tf*_*i*,*j*_ = number of occurrences of *i* in *j*, *d*_*i*_ = number of documents containing of *i*, *N* = total number of documents.

### Method

In our work, we compared 6 classifiers of supervised learning that learn and predict a categorical response that includes 4 categories as mentioned before. We adopted performance measures to assess the performance of classifiers, in particular accuracy, precision, recall and f1-score. We studied the performance measures of each classifier compared to all scientific papers. The performance measurement values reflect the careful selection of data from our database from the various scientific journals. We compared classifiers based on their respective best performance.

### Performance Metrics

In this subsection, we will focus on indicators that measure the quality of the model. To measure the performance of this classifier, we must distinguish 4 types of elements classified for the desired class namely: True Positive, False Positive, True Negative and False Negative. In the following, we present the performance metrics adopted to assess the performance of the different machine learning models used. Indeed, our assessment is based on 4 different measures including: Accuracy, Precision, Recall, F1-Score.

## Results

This section summarizes the experimental results obtained using our Dataset SPD/ED in two version, the extended and the closed one. In fact, we used several machine learning classifiers, aforementioned detailed. In each approach, the dataset is divided into train and test dataset with the ratio of 25% of test data and 75% of training data. Both train and test data need to be preprocessed and converted into feature vectors.

As depicted in Table 2, we present a comparative table of two approaches proposed at the level of this paper.

**Table 2.** Comparative table of machine learning algorithms based on our database in the case of 300 Full papers and 300 abstracts.

Machine learning methods	300 full papers				300 abstracts			
	Accuracy	Precision	Recall	F1-score	Accuracy	Precision	Recall	F1-score
SVM	**80%**	75%	81%	78%	**81%**	74%	72%	73%
KNN	**62%**	53%	58%	55%	**65%**	49%	56%	52%
RF	**81%**	85%	72%	78%	**83%**	81%	70%	75%
MN_NB	**74%**	82%	58%	68%	**79%**	83%	58%	69%
DT	**86%**	79%	86%	82%	**75%**	65%	74%	69%
GB	**78%**	81%	78%	79%	**81%**	75%	69%	71%

From Table 2, we can see that each algorithm shows high performance. In another side, we can see, from Table 2, that using the abstract (Aims, Methods, Results and Conclusion) only from the whole paper, is more fruitful and efficient in terms of accuracy. Then, we conclude that SVM, RF and GB are more accurate than the others used algorithms.

Based on the experimental results, we first concluded that the best scores obtained are justified by the relevant choice of scientific papers in the learning phase. Second, we can see, according to Table 2, that the training data process with the Abstract approach is more efficient and fruitful in terms of accuracy than the Full paper approach. We recorded that SVM, KNN, RF and MN Naïve Bayes present motivating performances.

We explored both methods: Machine learning and Deep learning. In contrast to image classification, we observed in our specific application of text mining that the time-consuming process of deep learning does not outperform machine learning. At the contrary, in some cases, machine learning produces significantly better results. We believe that, in our experience, deep learning does not provide efficient results given the size of our database.

## Conclusion

In this paper, we presented a comparative study of two different approaches of text classification using supervised machine learning classifiers. We started by identifying different methods of machine learning for text classification. Based on the literature review, we presented a survey on the machine learning techniques proposed for text classification. Through extensive experiments, we evaluated 6 methods based on our proposed dataset in the epidemiological domain. To the best of our knowledge, this is the first comparative study on scientific papers classification in the epidemiological domain. We proceeded with a careful selection of the different scientific papers, was made, based on a list of predefined classes according to the taxonomy of the epidemiological studies including: descriptive, analytical experimental and meta-analysis. Based on our experimental results, we emphasize that the learning done on the Abstract part (Introduction, Methods, Results, and Conclusion) is much more efficient than working with full paper because the divergence of the subject in question.
